# *“Es Lindo, Pero Estamos Perdidos”—*Characterizing Facilitators and Barriers to Clinical Genetic Testing for Latino Populations

**DOI:** 10.1007/s40615-025-02432-7

**Published:** 2025-04-15

**Authors:** Daniel Chavez-Yenter, Jeannette Villalta, Kimberly Kaphingst

**Affiliations:** 1 Division of Hematology-Oncology, University of Pennsylvania, Philadelphia, Pennsylvania USA; 2 Department of Medical Ethics and Health Policy, University of Pennsylvania, Philadelphia, Pennsylvania USA; 3 ¡Vamos! Health, West Valley City, Utah USA; 4 Department of Communication, University of Utah, Salt Lake City, Utah USA; 5 Huntsman Cancer Institute, University of Utah, Salt Lake City, Utah USA

**Keywords:** Genetic testing, Latino, Spanish, Facilitators, Barriers, Primary care, Attitudes, Norms, Self-efficacy

## Abstract

As genetic testing in clinical settings has been increasingly implemented, Latino populations are not benefiting at the same rates. Although many Latino individuals express a strong interest to use genetic testing, significant disparities exist with uptake. This study used semi-structured qualitative elicitation interviews to identify relevant referents (network of family/friends), socio-environmental facilitators, and barriers to genetic testing for a Latino-only cohort. The interviews explored attitudes toward genetic testing; beliefs about genetic testing, social norms, and control over ability to have genetic testing; and confidence in ability to test while probing upon perceived barriers and facilitators. Interviews were conducted with 19 self-identifying Latino individuals older than 18 years of age who spoke English or Spanish, stratified by previous testing or none. Respondents had overwhelmingly positive attitudes towards genetic testing. Many noted they simply did not understand the purpose of testing, what results could mean, and what results could be used for. Further, related to this limited knowledge, many noted their primary care providers seldom mention genetic testing. Finally, language preferences seem to be a critical barrier, as all those who have tested reported receiving services in English. Despite positive attitudes, strong referent networks, and strong interest towards genetic testing uptake for respondents, many also mentioned they had no idea how to test. More outreach is needed to educate Latino groups, and their providers, about genetic testing, while creating more systemic facilitators in genetic testing service delivery to encourage testing uptake.

## Introduction

Genetic testing in clinical settings has grown exponentially and has transformed the field of personalized medicine [1]. Prior research shows that Latino populations often have a strong willingness to receive genetic testing but are limited with lower access to health care [2]. Lack of knowledge has also been frequently cited as another explanation for underutilization [3]. There are notable disparities with the uptake of various types of genetic testing for Latino groups compared to White populations, be it cancer predisposition testing, prenatal screening, or carrier screening [3–9]. This has led researchers to question why these differences exist, and specifically explore potential barriers and facilitators exist to genetic testing. Two primary levels of barriers have been identified, specifically relating to individual-level issues or systemic-level issues.

Much of the research on genetic testing for Latino groups has focused on investigating their individual-level attitudes and beliefs towards genetic testing or precision medicine [3, 4, 10, 11], while a smaller number have begun to investigate actual experiences with genetic testing [5, 12, 13]. Unfortunately, a persistent lack of awareness has been noted for Latino groups when it comes to genetic testing (cancer predisposition, carrier screening, prenatal/newborn screening) [3, 14–16]. Another barrier noted for Latino groups is the potential negative affective responses to a positive result of a gene mutation, especially when there are nonactionable results [5, 12, 13, 15]. Other commonly identified barriers are related to social determinants of health at the individual level, including education, lack of health insurance, economic constraints, and language preference [12, 13, 15, 17, 18].

Systemic-level barriers, including lack of insurance, access to specialists, and language barriers, have all been identified as drivers of genetic services disparities for Latino groups [16, 19–21]. Latino patients are more likely than White patients to have primary care providers less knowledgeable of genetic testing and who may not refer their patients for proactive testing or consult with a genetic testing provider [22–24]. Genetic services delivery may have additional barriers. Communication directly with the genetics provider is often a significant challenge, specifically with the provider being unable to directly communicate to the patient in Spanish [25, 26]. Spanish-speaking patients often need a medical interpreter for services, with miscommunications or incomplete translations as common problems [27–29]. Further, even when interpreters or bilingual physicians provide genetic testing services, often there are misinterpretations due to missing or incorrect genomic terminology during disclosure of exome sequencing results [30], and cancer genetic counseling [31].

Facilitating factors for genetic counseling and testing for Latino groups at the individual-level include benefits for family, desire to take proactive actions for cancer (or other conditions), and provider recommendation or referral [22, 32, 33]. Further, when Latino individuals have a better perception of the benefits of testing, greater willingness to share personal health information, fewer information needs, greater genetic literacy, and greater satisfaction with healthcare providers, they had a higher likelihood of testing [14, 15, 34, 35].

While all of these studies have been important for identifying and characterizing facilitators and barriers to testing, there are limitations. First, many of the studies noted above often focus on one type of genetic testing. While facilitators and barriers are likely distinct within each testing context, it is important to build a more robust understanding of facilitators and barriers that are salient across context and determine if some may be present (or absent) in predicting behavioral intention to testing. As such, this study used elicitation interviews to identify salient attitudes that key members of the Latino population hold concerning genetic testing across two contexts, as well as both system-level and individual-level facilitators and barriers.

## Methods

### Design

We used open-ended elicitation interviews based on the integrated behavioral model (IBM) to identify relevant referents (networks), facilitators, and barriers [36]. The IBM posits that any given behavior is likely to occur with a strong intention to perform the behavior, especially if one has the necessary skills and abilities to perform the behavior, and given there are no environmental (or other) constraints on performing the behavior [37, 38]. IBM includes four subordinate constructs of salient attitudes addressed in the semi-structured elicitation interviews: (1) experiential attitude/affect (positive and negative), (2) behavioral beliefs (positive and negative), (3) normative beliefs and referents (groups or individuals who would be in favor or oppose the behavior), and (4) control beliefs and self-efficacy derived from behavioral health theories [39–41].

Attitudes towards the behavior are defined as the person’s overall favorableness (or unfavorableness) towards performing the behavior. To better characterize attitudes, Fishbein [42] explicated upon the concept by including experiential attitude or affect, or the individual’s emotional response to the idea of performing a particular behavior. In other words, individuals with a strong negative emotional response to a behavior are unlikely to perform it. In contrast, a person with a strong positive emotional response is likely to engage in it.

Normative belief is a person’s subjective perception of whether referents (groups or individuals in the person’s network) approve or disapprove of performing the behavior, weighted by their motivation to comply with their referents. As such, others’ expectations and behaviors contribute to perceived norms, inclusive of injunctive and descriptive norms. In other words, perceived norms reflect the social pressure one feels to perform or not perform the behavior.

Control beliefs (synonymous with personal agency as described by Bandura [43]) reflect self-efficacy and perceived control. Perceived control is one’s perceived amount of control over a behavior, determined by one’s perception of environmental factors that could make it easy or difficult to perform the behavior. On the other hand, self-efficacy is one’s degree of confidence in the ability to perform the behavior in the face of particular obstacles or challenges. It is important to note the difference here that perceived control refers more to environmental factors, such as transportation, access, and other factors that could be barriers to testing. Self-efficacy could relate to one’s confidence of being able to perform the behavior despite all the obstacles. Leveraging the IBM as a model allowed us to build robust semi-structured elicitation interview questions inclusive of normative or environmental facilitators and barriers.

#### Recruitment

We conducted interviews with 19 self-identifying Latino adults 18 years and older who spoke English or Spanish, stratifying the sample as nine individuals who had genetic testing for any health purpose and ten who had not, as recommended by elicitation guidelines [36]. The rationale for stratifying the sample by genetic testing was to be able to identify the differentiating factors for the behavior and which may be most salient and prone for intervention. We reached saturation at 19 participant interviews consistent with previous research using conceptual elicitation with the IBM [36, 44–47].

We used the resources of the University of Utah Health (UHealth), Huntsman Cancer Institute (HCI), and the Hispanic Health Care Task Force (HHCTF) of Utah to recruit participants. Flyers in both in English and Spanish were created with the eligibility criteria, purpose of the study, and contact information for the participation. A *promotora* from the HHCTF first shared these flyers and identified community members interested in participating. To identify more individuals who had genetic testing, we contacted those who had previously had genetic testing at HCI and had given permission to be contacted about research studies. We sent email invitations with the flyer and recruited participants interested in participating. Interviews were scheduled in-person or by phone, based on participant preference. The protocol was approved by the University of Utah IRB.

#### Procedure

Once participants arrived for an interview, they completed the consent process with the first author in either English or Spanish, and signed a consent form. We then started each interview by asking participants their conception of genetic testing and then working from that framework (what types of genetic testing they are most familiar with and providing definitions or clarification when needed), which was effective in our prior work [32]. All participants were asked about their salient attitudes, facilitators, and barriers (see Table 1).

**Table 1:** Genetic testing elicitation questions by construct

Construct	Elicitation questions
Experiential attitude (attitude)	• How do you *feel* about the idea of genetic testing*? • What do you *like/dislike* about genetic testing?
Instrumental attitude/behavioral beliefs (attitude)	• What are some of the *benefits* that might result from genetic testing? To you? To your family, community? • What are some of the *negative effects* that might result from genetic testing? To you, your family, community?
Normative influence (facilitators and/or barriers)	• Who would *support* you getting genetic testing? Why? • Who would be *against* you getting genetic testing? Why?
Perceived control (facilitators and/or barriers)	• What things would make it/made is *easy* for you to get genetic testing? • What things would make it/made it *hard* for you to get genetic testing?
Self-efficacy (facilitators and/or barriers)	• If you want to get a genetic testing, how *certain* are you that you can? • What kind of things would help you overcome any *challenges* to get genetic testing?

^*^Genetic testing will reflect specific type for those who tested

Interviews were guided by a semi-structured protocol but remained flexible depending on the flow of the interviews, as long as all topics were covered [48]. Basic demographics were collected verbally at the start of the interview, including age, education, language proficiency in English, and subethnicity. Interviews ran from 30 to 90 min. Upon the end of the interview, participants were emailed a $25 gift card as thanks for their time. All interviews were transcribed from recordings. Spanish-language interviews were transcribed in Spanish.

#### Qualitative Analysis

We conducted a combined content and thematic analysis for the interview data [44, 48]. Codes were organized by parent constructs from the IBM, salient attitudes, referents, facilitators, and barriers related to genetic testing. Subconstructs were coded from three parent domains: attitudes, perceived norms, and perceived agency. Environmental and interpersonal facilitators and barriers fell within each of these domains; however, they were coded as a parent code to better differentiate from other subconstructs. We utilized salient attitude constructs, including experiential attitude/affect (positive and negative), behavioral beliefs (positive and negative), normative referents, control beliefs and self-efficacy, and environmental and interpersonal barriers as parent codes. The coding schema was deductive based on the IBM and sought to identify frequent key constructs within the IBM while using the terminology and words of the target population from the interviews. The thematic analysis further allowed us to inductively code constructs or themes that may not fit into one of these domains. Saturation in responses began to emerge around the 15 th interview with common themes of barriers: facilitators and attitudes were identified for both those who tested and who had not. All coding and analysis were completed by the first author with final interpretation being agreed upon by the full research team. We used NVivo version 10 for data analysis. Spanish-language transcripts were analyzed in Spanish and then compared with English findings.

## Results

### Study Participants

Nineteen participants completed an interview. As shown in Table 2, participants ranged in age from 35 to 67 years old (mean age = 47.2 years old); the majority were women (*n* = 16 women). All self-identified as Latino/a/e/x, with Mexican ethnicity identity most prevalent (*n* = 11). The majority of participants self-reported they were bilingual in English and Spanish (*n* = 12), and had a university or graduate degree (*n* = 14). Ten of the participants had not had previous genetic testing, and nine had prior genetic testing. Cancer predisposition testing (CPT) was most common (*n* = 7). Thirteen interviews were conducted fully in Spanish, while six were conducted in English.

**Table 2:** Study cohort demographics

Variable	*n* (%)
Age (mean)	47.2
Gender	
Female	16 (84.2%)
Male	3 (15.8%)
Subethnicity	
Mexican	11 (57.9%)
Puerto Rican	3 (15.8%)
Guatemalan	2 (10.5%)
Peruvian	1 (5.3%)
Argentine	1 (5.3%)
Venezuelan	1 (5.3%)
Education	
High school	5 (26.3%)
College	10 (52.6%)
Graduate	4 (21.1%)
Language proficiency	
Bilingual	12 (63.2%)
Advanced English	2 (10.5%)
Intermediate English	2 (10.5%)
Basic English	2 (10.5%)
Only English	1 (5.3%)
**Total**	***N* = 19**
Type of test used (*n* = 9)	
Cancer predisposition	7 (77.8%)
Carrier screening	1 (11.1%)
Prenatal screening	1 (11.1%)

The resulting analysis here will be divided into IBM conceptual anchors with subdomains and subthemes that emerged with exemplar quotes provided and translated when appropriate. When differences emerged between language preference, previous testing, testing type, or other, they are noted below.

### Attitudes

#### Experiential Attitudes

The vast majority of participants had very strong positive attitudes towards genetic testing and its use. Many noted they liked the idea of gaining knowledge from the test results for prevention purposes.

“I think it’s definitely awesome. It just gives you more of a preventative or idea of what preventative care you can do to avoid getting there…Knowing your history and the possibilities of you or your siblings or children having something, or why the importance of testing would be important in this case” (Participant 2, Female, English (EN), Not Tested)

“[It seems] like they can save a lot of lives…It can predict, it can give medicines and treatments. It can help save people who have these types of problems. For me, it’s a positive [thing].” (Participant 3, Male, Spanish (SP), Not Tested)

Many in fact, had little to say about the potential negatives about testing. However, both among those who had tested and those who had not tested, there were some who reported conflicting attitudes towards testing. When it came to potential results or results that may be too impactful emotionally, some noted that they would likely not want to know. However, even these respondents noted that they do see the value in testing and ability know in order to serve as a tool for prevention or preparation.

“Probably not [test] for cancer. Yeah, probably not…because I’ve seen what cancer does to a person. And I think just for picturing myself in that situation, I think is what makes it hard for me to want to know.” (Participant 5, Male, EN, Tested)

“It scared me [the test] to know the results of the mutation. But its good to know to be able to prevent and treat it. So all of this was new for me. They took a blood sample and I had to wait. Truthfully, I was scared the whole time waiting. The results came, and still scared me a bit, but I realized I could use the results to prevent and prepare based upon my genetic mutations.” (Participant 17, Female, SP, Tested)

#### Instrumental Attitudes and Behavioral Beliefs

When asked about perceived benefits to testing, many respondents viewed the technology as an opportunity for prevention and taking actionable steps towards a condition.

“I think the tests give us the opportunity to know ourselves internally…to give me information I can use to better understand my body…to know for example in my case if I have the gene for cancer, to know genes in other areas. I think it gives [also] knowledge to the population and more importantly the Hispanic community, information to better support searches for treatment, in exercise, in diet, etc., etc.” (Participant 7, Female, SP, Tested)

“It’s the ability to take back the knowledge. I would go with doctor, and have a map of things going on, and use the results to help and understand what to do.” (Participant 8, Female, SP, Not Tested)

Another benefit that many noted was of its ability to not only inform their own individual risk, but similarly that of their family members.

“For the family, of course! If I do the test and find a genetic risk in my gene, I can let others in my family know…It like expanding our knowledge, expanding into concrete knowledge if I do it. For my family and friends, I [think] it’s really good.” (Participant 10, Female, SP, Not Tested)

“The power to know and prevent disease. More on a positive timeline. That we can think about our risk and put our attention on ways to prevent it. And the ability to let our family know about our potential risk…if someone tests positive for some diseases or condition. Then we can communicate it to our family so they can also get these type of tests.” (Participant 7, Female, SP, Tested)

Participants tended to not really have any negatives attitudes towards testing, but when asked how other in their community, or network of family and friends, some had a few responses on how some people may react negatively to the tests.

“And so many people are like, oh, I don’t want to get tested. I don’t want to go get, you know, colonoscopy, mammogram, because that’s going to hurt. And what if this and what if that? And like if you can catch it early and live. That’s what it is. If you can catch it early, you can live. If you can’t, you don’t. You die. I mean, that’s the reality. So my feelings are, is that people should get tested.” (Participant 11, Female, EN, Not Tested)

“Like fear is, [it] has more power over education and the need to know and do preventative care services…That’s why they prefer not to have information out there or people reach out to them for research and or genetics. I think that’s what it is. They have fear. I don’t see that there’s going to be negative things. I think it’s going to help.” (Participant 12, Female, EN, Not Tested)

### Norms and Control

#### Normative Influence

Participants reported that the majority of the family and friends would support their use of either type of genetic test.

“I would say my friends, almost everyone. And of my family, I think everyone would support me getting one [a test]. Specifically, my family. Because we’ve always been on the side of science and education and because of that they would accept it.” (Participant 9, Female, SP, Not Tested)

“My husband, my children, my parents, my siblings…I can’t think of a person right now honestly [to be against testing].” (Participant 12, Female, EN, Not Tested)

Yet, some participants noted that some in their network may not be as quick to support them to test for various reasons.

“I would say my dad, he’s very, like I said earlier, like I consider myself like, religious, spiritual, but like, I’m also kind of open minded. But my dad is more religious to the extent of like he doesn’t believe in… how do I say, fundamental Christian beliefs where God controls everything and we’re just here to do God’s will, essentially.” (Participant 5, Male, EN, Tested)

“[My] daddy always had the naysayers. They, you know, go “no, no, no”. And its just the negative, the pessimist people. If that’s going to hurt you more, that’s going to do more damage. Why would you do that? You know? But if I were do genetic testing, it wouldn’t be for them. It would be for me. So they can say whatever they want.” (Participant 11, Female, EN, Not Tested)

For all those who had tested, none reported their family being against testing. In fact, many of those who tested said they did it as a result of their family history and wanted to know for themselves for their own medical care plans.

“I worried [about the result] because of my dad, he died of cancer. So the doctors told me that may be a high possibility that my sons and daughters could have that same genes for cancer too.” (Participant 1, Female, SP, Tested)

“I did the first genetic test when I was 39 years old, because my mom died at that exact age. At 39 she died of breast cancer, she was a nurse and never had a preventative exam and died when the cancer was very advanced. So at that age, I decided to take the test myself to see if I had the same predisposition to breast cancer, and I was open to either result.” (Participant 6, Female, SP, Tested)

#### Perceived Control

First and foremost, to make testing easier, every respondent said more information needed to be more readily available for themselves and/or others to better understand and know the purposes of testing.

“I’m pretty sure that the majority of the Hispanic community doesn’t have any knowledge of genetic testing and its benefits…then the need is for more information [is needed].” (Participant 13, Female, SP, Not Tested)

“Information. Information that can come from a brochure or a pamphlet, that has precise [information] about what genetic tests do or maybe at health fairs, promote these types of tests, and give more information to the community. Because that would better spread and inform people and more successful, no?” (Participant 9, Female, SP, Not Tested)

“I think to promote first, its necessary that they understand the power [tests] have for future generations.” (Participant 8, Female, SP, Not Tested)

When asked to expand on their answers, many suggested various ways to promote genetic testing information through a sense of marketing in ways that the Latino/Hispanic community would receive it.

“I think there’s a lot of lack of information. Like I told you, here in Utah, it’s a great place for doing [DTC] testing to know our ethnicities and they use a lot of publicity to promote that…but why aren’t we using marketing for our communities of this type of testing for health?” (Participant 7, Female, SP, Tested)

“The challenge is educating. Yes, it’s a challenge for groups, organizations, including churches, who open their doors to educate people…I think the education, being able to respond is very important to clear any doubts the person would have. I think it would be much better.” (Participant 3, Male, SP, Not Tested)

“I think there needs to be more announcements on the radio, television, social media, where we are seeing more younger women coming in to our organization with breast cancer in her 20 s” (Participant 14, Female, SP, Tested)

Similarly, when asked more about what barriers they believe exist with these suggested improvements, many of them noted the challenges of Spanish-language information about these tests being lacking in not only public spaces, but in clinical settings as well.

“I haven’t seen like Spanish materials or companies or anything about genetic testing other than Ancestry.com…language could potentially be a barrier, especially for people that don’t have like higher levels of education…even if you do translate them, will people understand what that word even means?” (Participant 5, Male, EN, Tested)

“I think we still have to do a lot for written Spanish language information, where a person can understand, for example, what you just said, the cancer screening genetic test isn’t just the only test.” (Participant 14, Female, SP, Tested)

“Its Spanish I know, what I see, what I write…Spanish is my primary language and I understand things better it in, even though I do understand things in English…I think in the worse of cases, genetic counselors who don’t speak Spanish, with the help of a translator, can make sure the community understands, but it may not cover everything. Just to make sure the person better understands [the topic].” (Participant 6, Female, SP, Tested)

To that end, many suggested even before a person would want to seek out the test, that there needs to be entry points with a person who can better speak the language and communicate the importance of the tests.

“We need people who speak the same language who can talk to our communities...And especially explain in their own terms the benefits of getting genetic testing done, but using words that the community can understand.” (Participant 14, Female, SP, Tested)

“Community leaders are really important to the community as they have their trust and if they use other terms, I think [education] will work with them. It will be similar to their success with promoting vaccines.” (Participant 7, Female, SP, Tested)

In these cases, it was notable that these were settings outside of the clinic, with more trusted community leaders or certain organizations that could take on the role of educators for genetic tests. However, some participants, including those who have tested, noted how their primary care providers filled this role.

“So I brought my questions to my provider, because I selected her, because I have to feel that my provider is at my service. If that provider is not at my service, then they won’t be my provider. So when she sat with me, and talked me through the process, and told me how easy it was, I felt I had all the necessary pieces of information to know and felt calm about what the test would be and the best possible result it would do to give advice to me and my daughters too for medical care.” (Participant 14, Female, SP, Tested)

“I see the doctors as important too. Its really important to see them in spreading the information of genetic tests, not just messages on chronic disease…I’ve never heard a doctor recommending a genetic test or they should, and they should explain the different potential results. Or at least if they don’t know, they should know where to find that information.” (Participant 15, Female, EN, Not Tested)

For those who did receive a genetic test, they reported their doctors were strong facilitators when they had strong relationships. Many liked the idea of doctors/provider directing the genetic testing processes to make the process easier.

“My doctor always wants to do up-to-date mammographs, because of the history of my mom, and she always told me it would be a good idea to do the genetic test to have certainty to know if I was a carrier. So it was then that I decided to do the test because my doctor referred me to another agency or place to do the test a couple of years ago. But it was the doctor who referred me and walked me through the process.” (Participant 6, Female, SP, Tested)

“I think it should be done before, like a standard practice for at least for women because we know that there’s a relation between, genetic, mutations and all that with breast cancer. So I think it should be done before diagnosis. Primary care physician or gynecologist should recommend that the way that they recommend, I don’t know, a colonoscopy.” (Participant 16, Female, EN, Tested)

However, a common challenge for those who tested and were Spanish-preferring, noted some challenges in translating results, or just receiving the results in English, even if they were limited in English or Spanish-preferring.

“The doctor offered me a translator. And I wanted one. I needed one even though I understand English, but with medical terminology, not as much. But she [the translator] was nice and she helped me in the moment better understand and translate what the doctor was saying.” (Participant 17, Female, SP, Tested)

“I arrived and they did my test right away and sent the blood sample to a lab in California…all in English, my doctor is American and doesn’t speak Spanish…and the results call was in English too.” (Participant 6, Female, SP, Tested)

Notably, this demonstrated that medical care systems with these types of resources could better serve the community for genetic testing service delivery. To do that effectively though, one respondent in particular noted, that if the tests were to really be more accessible to Latinx groups, then they needed to make them more readily accessible in community clinic settings.

“More resources are needed. More resources about whether these tests have certain costs and are accessible in community clinics…To see even if community clinics are order these types of tests in an accessible way that’s available for everyone. If not, then only some will be able to do it, and others won’t. If they could order them, then it would be better.” (Participant 7, Female, SP, Tested)

#### Self-Efficacy

When it came to asking participants how certain they are in being able to test, many of them reported limited confidence in being able to find ways to start the testing process.

“Everything is beautiful [about testing], but no. Where do I need to go [to get testing]? The majority of people aren’t going to know how to get testing done.” (Participant 8, Female, SP, Not Tested)

“No, I wouldn’t know where to go. Where? Or at the least my clinic, because when I go to the clinic or one reason or another. But in my mind that’s where I could ask about genetic testing.” (Participant 4, Female, SP, Not Tested)

When asked what would help them get genetic testing in their own circumstances, many of them noted they would want their healthcare providers to answer questions and offer them the potential referral to testing or to have testing on site to offer right away.

“I think the clinic would need to have the test, because it may be that people would not want to go to [another] hospital or something similar [to test]. Another thing that one doesn’t think about is cost, so for me that be inaccessible.” (Participant 4, Female, SP, Not Tested)

“I think if I ask my doctor about it what do I ask? What can I say? How open will my doctor for me to do the test? More than anything would she be supportive and give me the reference to actually do the test?” (Participant 13, Female, SP, Not Tested)

“Accessible. I know they need to be accessible. That’s how they asked me in the moment to test, they offered it [right away]. The primary doctor always directs it. The primary care doctors have to be the first connections with companies that do genetic tests.” (Participant 6, Female, SP, Tested)

Again, many participants believed if and when they decided to go ahead with testing, they would want their healthcare provider to offer testing and guide them through the process and answer questions that may arise, including if their insurance would cover the cost. The cost and whether insurance would cover testing was noted as another potential barrier to testing uptake.

“I wouldn’t know how to get a test. I don’t know how, how much economically it would cost or how easy it would be to do it. I would need to see about that. How to educate myself about what I have to do if I want it, what it would do, and what I would be submitting to.” (Participant 3, Male, SP, Not Tested)

“I want more information. To know how much it would be without health insurance. Because I’ve heard from people that have asked me about my own testing how much it cost and I respond and say that my insurance covered it, they still want to know the cost.” (Participant 6, Female, SP, Tested)

## Discussion

Overall, respondents had mostly positive attitudes and positive normative influence toward testing and were interested in testing; however, when it came to seeking out testing, many noted that they would have no idea how to start the process. For those who had tested, a key facilitator was their provider directing the process, suggesting an intervention may need to directly involve providers to promote genetic testing service delivery. Other notable barriers from respondents included language access, lack of information, and financial concerns for the cost of the test. Our results are consistent with previous research, but expands upon Latino respondents’ ideas on how to overcome barriers and leverage facilitators for testing uptake.

Attitudes toward testing are largely positive, but are seemingly influenced by low awareness among Latino communities [10, 16, 49–51]. Many Latino individuals report high interest in testing [16, 50, 52, 53], consistent with the findings from this study of respondents wanting more information. More efforts have been put forth for increasing knowledge of genetic testing for Latino populations through developing culturally relevant materials, promoting provider conversations and referrals, and using *promotores* to fill information gaps [14, 21, 27, 54, 55]. Some have begun to use *promotores* to provide culturally appropriate care and be inclusive of the wants and needs of the community when it comes to the service of genetic testing and counseling and informational needs in the context of breast cancer [14, 21]. Other researchers have begun exploring integrating genomic screening into primary care among Latino populations to ensure the technology can be available for patients who want to test [19]. Further, return of results processes have been developed to be culturally appropriate and responsive to ensure providers consider nonbiological factors that may exacerbate a positive result and how to further navigate and provide recommendations for care [56, 57].

From our interviews for both testers and non-testers, there was a desire for more information directly from their providers. Despite some studies emphasizing patient-level barriers for genetic testing for Latino communities including low knowledge of genetics and numeracy [51, 58–60], another notable system-level barrier is low referral rates from primary care providers for Latino patients [17, 20, 22, 23]. Low referrals rates may stem from many factors, including challenges of patient-provider communication, especially with language differences, collection of family health information, and unfamiliarity with reimbursements/costs for genetic testing [22, 61–63]. Interviewees who had tested commonly mentioned they needed to prompt their provider by requesting genetic testing, rather than getting information and a referral from the provider. If providers are not providing referrals and Latino patients have low awareness of testing, they likely will have low perceived agency/control in seeking out testing. There are recent initiatives being used to try and integrate genetic testing in primary care [13, 19, 56]; however, these remain pilot initiatives within only one healthcare system and need further expansion to address these referral disparities.

For those who did test, their provider recommendation or referral was a key facilitator for them seeking out testing. Patient-provider communication approaches that acknowledge competing demands, emphasizing familial connection, and individual well-being can promote genetic testing engagement [51, 64, 65]. Yet, to achieve this, interventions need to be tailored to the Latino community, especially if they are Spanish-preferring. Integrating particular communication strategies have shown success, such as avoiding hypotheticals, using simpler terminology, teach back strategies, and adapting results communication based upon psychosocial profile of the patient [31, 66].

However, a key challenge remains for providing services for Spanish-preferring patients, namely the language barrier. From our interviews, healthcare system resources for translation and interpretation are a key factor. Leveraging interpreters or *promotoras* can help build a cultural and/or informational bridge between providers and patients [51, 53, 67]. However, some systems do not have resources like interpreters or *promotoras*, highlighting a key barrier for those in areas with limited language resources. Recent initiatives have been developed to train bilingual *promotoras* to fill the information and cultural gap by educating patients on hereditary breast and ovarian cancers for Latina women [21]. Pilot results show promise for this approach to identify and refer high-risk Latina women, highlighting that this approach can work, but needs more resources and effort dedicated to these types of interventions.

As with any qualitative study, our results cannot be generalized for the larger Latino community in the United States. The Latino community is highly diverse containing multiple subethnicities and racial compositions [68]. More stratified research by subethnicity could yield important results, particular around shared decision making. Second, our cohort was fairly homogenous being well educated, mostly bilingual, and having previously used cancer predisposition testing. As these individuals may be more acculturated, their experiences, attitudes and perspectives may differ from those less acculturated, as seen in the existing literature [51, 69, 70]. Lastly, for those who had used genetic testing, there is a possibility of bias, as many reported positive experiences. Still, for those who had tested, there were things they believed could be improved and were willing to share. We uncovered influential facilitators and barriers we believe would be present regardless of acculturation status, like provider referral, clinic resources, and normative influence.

Our findings appear consistent with the IBM, with attitudes towards genetic testing, normative influence, perceived facilitators and barriers, and agency all potential influences on testing. Attitudes towards testing were largely positive; however, for those who had not testing, many needed a definition of testing provided before they could make an evaluative judgement on testing. Positive attitudes can be a facilitator, but a lack of knowledge of testing appears to be biasing attitudes towards testing as a current barrier. Normative influence seemed to indicate that family and friend networks could be important facilitators to testing, rather than barriers, as many believed their networks would have positive attitudes towards testing. Perceived agency appeared to be the biggest barrier to testing for respondents. Many reported a desire for their primary care doctor to direct the process or provide a referral to testing. For those who had tested, many noted that their provider directing the process was a key facilitator. This highlights an important finding outside of individual perception yet has important implications. Interventions to improve agency will require clinical-level initiatives. Thus, targeted interventions based on these theoretical foundations do appear to indicate that each domain can be targeted for improvement. For each intervention developed, tailored and targeted materials should be considered within the context of the desired outcomes. With those considerations, interventions can truly be impactful in reducing inequities relating to genetic testing.

## Data Availability

Data is available from the authors upon request. Contact Dr. Daniel Chavez-Yenter for data inquiries.
